# Physical Activity Interventions for Postnatal Weight Management: A Systematic Literature Review

**DOI:** 10.21315/mjms2023.30.6.5

**Published:** 2023-12-19

**Authors:** Siti Zuhaidah Shahadan, Siti Juliana A. Rahman, Mohamad Firdaus Mohamad Ismail, Nurvita Risdiana

**Affiliations:** 1 Department of Medical-Surgical Nursing, Kulliyyah of Nursing, International Islamic University Malaysia, Pahang, Malaysia; 2 Institut Latihan Kementerian Kesihatan Malaysia (Kejururawatan), Negeri Sembilan, Malaysia; 3 Department of Professional Nursing Studies, Kulliyyah of Nursing, International Islamic University Malaysia, Pahang, Malaysia; 4 School of Nursing, Faculty of Medicine and Health Sciences, Universitas Muhammadiyah Yogyakarta, Yogyakarta, Indonesia

**Keywords:** physical activity intervention, postnatal women, weight management, postnatal weight retention, obesity

## Abstract

Physical activity (PA) maintains weight and reduces postnatal weight retention (PWR), thereby lowering obesity-related comorbidities. There is only limited evidence on the most effective postnatal PA for Malaysian women. This review identified evidence-based literature on the effectiveness of PA interventions in managing weight in postnatal women and the preferred type of intervention for them. A systematic literature search was conducted following the preferred reporting items for systematic reviews and meta-analyses guidelines. Randomised controlled trials and quasi-experimental research related to PA interventions for women during the postnatal period (18 months after delivery) published in English from 2011 to 2021 were searched in ProQuest, Scopus, Springer Link and PubMed using the following keywords: ‘physical activity’, ‘weight management’ and ‘postnatal women’. Intervention groups with weight and body mass index measured after any supervised PA guidance/counselling with a minimum follow-up of 10 weeks were included in the analysis. Those with pharmacological management and a comparator control group were excluded. A total of six articles met the inclusion criteria. Using the revised Cochrane risk of bias tool for randomised trials, one of these articles was classified as low-risk, two as having some concerns and three as having a high risk of bias. Walking, yoga and Pilates were found to be the most appropriate and preferred types of physical activity, despite having a small but significant impact on postnatal women’s weight management. Healthcare professionals should adopt programmes that explicitly target these PA interventions to manage PWR.

## Introduction

The number of overweight people is increasing worldwide and the condition can strongly contribute to obesity later in life (1). Postnatal weight retention (PWR) is one of the top contributors to the development of obesity after pregnancy (2). The factors contributing to PWR, such as the high body mass index (BMI) during pregnancy and excessive gestational weight gain, vary greatly for each pregnant woman (2). Average PWR ranges from 0.5 kg to 3 kg; however, it varies greatly and up to 20% of women hold weight greater than 4 kg at one year postnatal (3). Strategies that can prevent PWR include balanced diet and physical activity (PA). Specific methods have not yet not been investigated in the postnatal weight management literature.

This review discusses PA interventions for managing weight in postnatal women. In a Malaysian setting, there are currently no specific documented clinical practice guidelines regarding PA in managing weight during the postnatal period, even though many interventions have been introduced globally (4). Nevertheless, it is widely accepted that PA is a therapeutic modality with benefits such as maintaining weight and reducing PWR after childbirth, thereby reducing the incidence of obesity-related comorbidities, which include cardiometabolic diseases. Randomised controlled trials (RCTs) and systematic literature reviews (SLRs) have demonstrated that PA effectively manages postnatal weight after childbirth (4, 5). Specifically, Ferguson et al. (6), in their SLR, revealed that lifestyle interventions that involve diet and physical activity, physical activity or diet alone significantly reduced weight in postnatal women by 1.7 kg (95% CI: −2.3, −1.1) relative to the comparator at follow-up. Tyldesley-Marshall et al. (7) found various types of PA for postnatal weight management. However, high levels of attrition and poor engagement in postnatal women have been identified as issues, and more attractive and attainable yet flexible approaches are recommended (3). Therefore, this systematic review aims to identify the evidence-based literature on the effectiveness of PA interventions in managing weight in postnatal women and to identify the most appropriate and preferred PA interventions for this group. Specifically, the participants, interventions, comparator, outcomes and study design (PICOS) of the review were any PA interventions for women during the postnatal period (18 months after delivery), where 18 months postnatal was chosen because it is the longest period base on the preliminary reading of the literature; any supervised PA guidance/counselling with a minimum follow-up of 10 weeks; any comparator group as a control group with minimal intervention, usual care for the given study setting or delayed intervention; the changes in either body weight or BMI from study initiation to completion, and RCT and quasi-experimental study only, respectively.

## Methods

The reporting in this review was carried out according to the preferred reporting items for systematic reviews and meta-analyses (PRISMA) for protocols 2015 guidelines, and PICOS search tools, which include participants, interventions, comparators, outcomes and study design, were utilised.

### Search and Data Sources

The inclusion criteria for this review were based on either the PICOS or the participants involved, type of intervention, type of comparison group, outcomes of interest and study design (Table 1). The exclusion criteria were PA interventions that were integrated with pharmacological interventions. The search included both published and in-press articles. Databases including ProQuest Health and Medical Complete, Scopus, Springer Link and PubMed were used for the search. The year of publication was filtered to between 2011 and 2021, and the language of the articles was filtered to English. The following keywords are used for the search: ‘physical activity’, ‘weight management’ and ‘postnatal women’. Any related text words and synonyms from the keywords by MeSH search terms were also used. The Boolean operator ‘AND’ was used to limit the search by combining each keyword, and the Boolean operator ‘OR’ was used to expand it and fetch more mentions of each keyword.

### Study Selection

The selection of studies began by screening the titles and abstracts for inclusion and exclusion criteria. Two reviewers (the first and second author) worked independently to identify original studies in the literature lists that were eligible for further review by screening abstracts and titles. If a study was determined to be relevant, the full-text manuscript was obtained and reviewed. Information was extracted to a standard survey tool explicitly created for this systematic review. Publication date, study details, participant characteristics, intervention characteristics, self-report and objective PA outcome measures and other pertinent information were all collected from the selected studies. Only journal articles with full text available were chosen. Other than that, the reference lists of the studies included for analysis were reviewed to identify any additional studies that might have been eligible. All duplicated records were screened and removed. Once the screening process was complete, the data were entered into the PRISMA flow diagram. The revised Cochrane risk-of-bias tool for randomised trials was used to assess the quality of the chosen articles (Table 2). This tool is used to evaluate five different types of bias, namely bias from the randomisation process, bias due to deviations from intended interventions, bias due to missing outcome data, bias in the measurement of the outcome and bias in the selection of the reported result. Answer options within each domain included ‘yes’, ‘probably yes’, ‘probably no’, ‘not applicable’ and ‘no information’. Judgement of the overall bias of the articles was then made based on the responses from each domain and were ranked as ‘low risk of bias’, ‘some concerns’ or ‘high risk of bias’. All included articles were evaluated for bias by the first and second authors.

## Results

**Figure 1:**
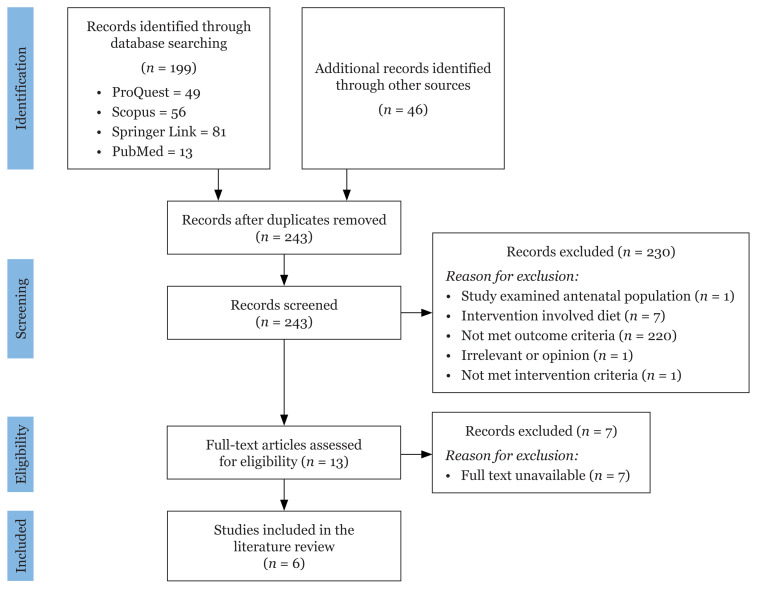
PRISMA systematic review flow diagram for screening and selection of identified studies

The search strategy of this review was based on the PRISMA 2009 flow diagram (Figure 1). The number of records identified, included and excluded and the reasons for exclusions were mapped out through the different phases of the search. There were no restrictions on the type, frequency, duration or intensity of the PA intervention. Interventions not explicitly designed to target weight measurement were excluded. The outcomes were the mean difference of either body weight or BMI. A total of 243 studies were returned from the four electronic databases, hand-searching and a bridge search. After screening, six studies were identified that were related to PA intervention for weight management in postnatal women, and they were included in this review.

### Study Characteristics

The studies consisted of five RCTs and one quasi-experimental design study. They were conducted in the USA, China, Iran, Australia and Greece, with four studies recruiting participants through a community-based (8–11), one study from an outpatient clinic (12) and one from a health clinic (13). Among the six studies included in the analyses, 629 participants were randomised to intervention (*n* = 302) and control (*n* = 299) groups. The age range of the participants was between 18 years old and 45 years old. With regard to the duration of the postnatal period, two studies included postnatal women between 6 weeks and 6 months (9, 13), one study each included postnatal women between 4 weeks and 6 weeks (10), 4 weeks and 6 months (12) and 8 weeks and 12 months (8). In a review for parity among the postnatal women, two studies specified primiparous to multiparous (8, 11), one recruited only primiparous (10) and three did not report this element (9, 10, 13). Meanwhile, the postnatal women included in the analysis had normal to obese (BMI 18.5 kg/m^2^–40 kg/m^2^) (8), overweight to obese (BMI ≥ 25 kg/m^2^–35 kg/m^2^) (9) and normal BMI levels before pregnancy (BMI 18.5 kg/m^2^–24.9 kg/m^2^) (13). Three studies did not report the BMI levels of postnatal women (10–12). Concerning breastfeeding practice eligibility criteria, only two studies included breastfeeding women (10, 13), two had no restriction (8, 12) and two did not report any of the breastfeeding criteria (9, 11).

### Main Findings

#### The effectiveness of PA interventions in managing weight in postnatal women

Based on the findings, five studies took BMI readings to measure anthropometric outcomes (8, 9, 11–13) and one study only measured weight (10). The study by Albright et al. (8) using an intervention with 10,000 steps per day individually for 18 months showed significantly increased PA levels, but the changes in BMI were not reported. Meanwhile, the study by Keller et al. (9) used interventions with 30 min–150 min of PA per session every day individually for 12 weeks, which showed significantly reduced BMI. The study by Kernot et al. (11) using a PA intervention group walking exercise with a target of 10,000 steps a day for 50 days showed that BMI in the participants significantly decreased. The study conducted by Maturi et al. (13) on free-living step exercises with a target of 10,000 steps at the end of 12 weeks found a significant reduction in BMI. Moreover, the study by Ko et al. (12), which used yoga and Pilates as PA interventions in managing the weight of postnatal women for 60 min per session for one day per week over 3 months, showed that respondents’ BMI significantly reduced. In contrast, the study conducted by Zourladani et al. (10) using aerobic strengthening and stretching performed for 50 min–60 min daily three times a week found no significant weight reduction.

#### The preferred PA interventions for postnatal women

Most studies in this review chose walking exercise as their strategy of PA intervention in weight management in postnatal women (8, 9, 11, 13). One study chose yoga and Pilates (12), and one chose aerobic, strengthening and stretching exercise (10). The average length of the intervention was 12 weeks (9, 10, 13); one intervention lasted 50 days (11), another for 3 months (12) and the longest period was 18 months (8). In general, the intervention delivery method was classified as individual or group. Four studies used the intervention delivery method by group (9–12) and two studies were individual (8, 13). Meanwhile, in terms of supervising the participants, three studies’ primary delivery method was distance (8, 11, 13) and three were done face-to-face (9, 10, 12). A summary of the intervention protocols and findings of the included studies is provided in Table 2.

Preferred PA may also indirectly be monitored by intervention adherence. All studies reported some form of adherence-related or withdrawal information. Interventions associated with the highest adherence rates were where the programme had the highest compliance rate (100%) and all participants could join the programme until the end (8). The programme offered by Maturi et al. (13) had the second highest compliance rate (91.43%) and BMI was found to significantly reduce (*P* = 0.001). This was followed by Zourladani et al. (10)’s programme, which had the third highest compliance rate (90.90%), with 20 out of 22 participants finishing the programme, although their weight is not significantly reduced. The programme by Ko et al. (12) had the fourth highest compliance rate (82.14%) and identified significantly reduced BMI. Kernot et al. (11)’s programme had the fifth highest compliance rate (75%) and showed significantly reduced BMI, while the programme by Keller et al. (9) had the lowest compliance rate (54.92%), yet the participating postnatal women showed a significant reduction in BMI measurement. The reasons for low compliance or adherence rates included the dose of the PA (running), being too busy or lacking time, husband not allowing participation, transportation and childcare.

#### Implications for Practice

The purpose of this analysis was to identify the preference for and effectiveness of PA interventions in weight management in postnatal women, and its findings can be recommended as part of clinical practice guidelines. Based on a limited number of studies and including a few studies with high-quality evaluations containing RCT research designs, the overall findings suggest that walking targeted at 10,000 steps daily, yoga and Pilates have a small yet significant effect, thus they are appropriate and preferred types of PA intervention for weight management in postnatal women. Of the six identified studies, three found significant changes in reducing BMI after a period of PA intervention programmes (9, 11, 13). Nonetheless, several factors may have influenced the effectiveness of the PA intervention programmes, including the protocol used, the diverse delivery method and support strategies employed and the measuring procedures used to assess weight. Regardless, this review found that the PA intervention programmes were mainly implemented through social support, including motivation, encouragement and follow-up.

The findings of this review also suggest that PA interventions are generally successful in reducing the weight of postnatal women, albeit with most effect sizes being small. Individual or group supervised and structured (weekly frequency, scheduled durations and moderate intensity) PA intervention sessions that adhere to specific PA guidelines over an extended postnatal period (more than 12 weeks) are most likely to be associated with meaningful weight or BMI reduction. Furthermore, due to time constraints in managing daily routines and the newborn, the findings suggest that the type of PA intervention to be offered should be appropriate to the situations of individual postnatal women. This is supported by the findings from a qualitative study by Teh et al. (14), who found that postnatal women complained of fatigue, exhaustion and lack of self-care during the postnatal period, thereby limiting their willingness to engage in any PA interventions that seemed inappropriate to them. Pal et al. (15) also stated that walking is a preferred PA intervention for weight management in postnatal women due to its natural low impact and its low risk of injury.

Besides walking as the preferred and effective PA intervention, yoga and Pilates may be able to manage postnatal weight. Specifically, yoga addresses movement for meditation, and Pilates is more focused on muscle relaxation, strengthening and improving posture, and both PA interventions can be initiated as early as 72 h to 1 week after childbirth, as they do not involve vigorous movement (16). According to Timlin and Simpson (17), yoga and Pilates can help to restore a postnatal woman’s body to a pre-pregnancy state and can be done alone at home. However, because these activities involve specific movements, they require training by a trained instructor before they can be done alone at home. Nonetheless, evidence for yoga and Pilates in postnatal weight management is limited, since most studies focused on their effects on stress reduction and sleep improvement. As a result, more robust experimental studies are needed to demonstrate the effectiveness of yoga and Pilates in managing postnatal women’s weight.

This review found that most participants in the intervention groups complied with the PA due to factors such as follow-up strategies and social support. Among the strategies used in the studies selected in this review were weekly phone or email reminders and engagement with professional instructors. On the other hand, the reason for withdrawal from the PA interventions was a reduction in social support within 12 weeks of the postnatal period. Ellis et al. (18) affirmed that social support is necessary for motivating postnatal women to engage in regular PA to control their postnatal weight. Besides, establishing a support group, particularly within their social circle, can strengthen postnatal women’s support network and encourage compliance regardless of the intensity of the PA intervention (19). In short, regular weight management follow-up with healthcare personnel may encourage postnatal women to participate in PA. Aside from that, the findings of this systematic review suggest that social support networks, such as husband, family and friends, should always be involved in PA interventions designed for postnatal women.

## Conclusion

The current analysis discovered that PA interventions including walking, yoga and Pilates have a minor but substantial effect on postnatal weight management. More experimental trials with a bigger sample size are needed, however, to demonstrate the effectiveness of these PA treatments in managing postnatal women’s weight.

### Strength and Limitation

The strength of this review is that the nutritional component was excluded to obtain a clear picture of the specific PA intervention targeted for weight management in postnatal women. Nonetheless, the majority of included studies mentioned their small sample size, limiting the generalisability of the findings to a large population.

## Figures and Tables

**Table 1:** Inclusion and exclusion criteria for the literature searching process

Search tool	Inclusion criteria	Exclusion criteria
Participants	Postnatal women at any point up to and including 18 months after normal delivery	Postnatal mother with cognitive impairment or other medical illness, instrumental and operative delivery and antenatal mother
Interventions	Any types of postnatal exercise/physical activity	Diet intervention, heat therapy, pharmacological intervention and traditional practices
Comparators	An active or non-active control group	–
Outcomes measure	Body composition measures: weight and BMI	–
Study design	RCTs, quasi-experimental control trials	–

**Table 2:** The intervention protocol, study outcomes and risk of bias of the included studies

Author /Year	Participants and sample size (*N*)	Intervention protocol				Comparison group	Study outcomes	Risk of bias					Overall risk of bias
	
		Types of PA	Delivery strategies	Study design and length	Follow-up			Randomisation	Deviations from intended interventions	Missing outcome data	Measurement of the outcome	Selection of the reported result	
Albright et al. 2012 (8)	Postnatal women from Hawaii *N* = 278	Walking (10,000 step/day for 7 days/week)	One-to-one tailored telephone counselling *n* = 138	RCT 18 months	Health educator-initiated telephone or email contact over 12 months	Print/website materials *n* = 140	Weight and BMI were measured but no data was reported	Low risk	Low risk	Some concern	Low risk	Low risk	Some concerns
Keller et al. 2014 (9)	Postnatal women from Mexico *N* = 139	Walking (30 min–150 min/session for 1 day/week)	Group walking led by community health advisors *n* = 71	RCT 12 weeks	Weekly telephone contact	Newsletters and weekly telephone calls (unrelated to PA) *n* = 68	No significant difference in body weight between-group (*P* = 0.609)	Some concern	Low risk	Low risk	Low risk	Low risk	Some concerns
Zourladani et al. 2015 (10)	Postnatal women from Greece *N* = 44	Aerobic strengthening and stretching (50 min–60 min/day for 3 days/week)	Group session *n* = 20	RCT 12 weeks	Every three days per week of exercise by a certified instructor	No training programme at all *n* = 17	No significant differences in body weight between-group (*P* > 0.05)	High risk	Low risk	Low risk	Low risk	Low risk	High risk
Kernot et al. 2018 (11)	Postnatal women from Adelaide *N* = 120	Team-based walking (10,000 step/day)	Group-based walking exercise via Facebook *n* = 41	RCT 50 days	A weekly email, Facebook notifications and daily physical activity tips	Alternative intervention received pedometer only (*n* = 39), Control group that received written advice through email (*n* = 40)	No significant differences in BMI between groups (*P* = 0.30)	High risk	Low risk	Low risk	Low risk	Low risk	High risk
Ko et al. 2013 (12)	Postnatal women from Taiwan *N* = 28	Yoga and Pilates (60 min/session for 1 day/week)	Group session led by a professional instructor	Quasi-experimental 3 months	Weekly exercise sessions by professional instructors	No control group	Significant reductions in body weight within the group (*P* < 0.001)	High risk	Low risk	Low risk	Low risk	Low risk	High risk
Maturi et al. 2011 (13)	Postnatal women from Iran *N* = 66	Walking (Free-living step of 10,000 steps at the end of 12 weeks)	Follow-up individual session *n* = 32	RCT 12 weeks	Reminder and tailored phone text and health education pamphlet	Routine postnatal care *n* = 34	Significant differences between-group in weight (*P* = 0.001) and BMI (*P* = 0.001)	Low risk	Low risk	Low risk	Low risk	Low risk	Low risk
